# Advances in Cancer Diagnosis: Bio-Electrochemical and Biophysical Characterizations of Cancer Cells

**DOI:** 10.3390/mi13091401

**Published:** 2022-08-26

**Authors:** Kholoud K. Arafa, Alaa Ibrahim, Reem Mergawy, Ibrahim M. El-Sherbiny, Ferdinando Febbraio, Rabeay Y. A. Hassan

**Affiliations:** 1 Nanoscience Program, University of Science and Technology (UST), Zewail City of Science and Technology, Giza 12578, Egypt; 2 Institute of Biochemistry and Cell Biology, National Research Council (CNR), Via P. Castellino 111, 80131 Naples, Italy

**Keywords:** cancer biology, metastasis, apoptosis, in-vitro assessment, electrochemical biosensors, electron microscopy, atomic force microscopy

## Abstract

Cancer is a worldwide leading cause of death, and it is projected that newly diagnosed cases globally will reach 27.5 million each year by 2040. Cancers (malignant tumors), unlike benign tumors are characterized by structural and functional dedifferentiation (anaplasia), breaching of the basement membrane, spreading to adjacent tissues (invasiveness), and the capability to spread to distant sites (metastasis). In the cancer biology research field, understanding and characterizing cancer metastasis as well as features of cell death (apoptosis) is considered a technically challenging subject of study and clinically is very critical and necessary. Therefore, in addition to the cytochemical methods traditionally used, novel biophysical and bioelectrochemical techniques (e.g., cyclic voltammetry and electrochemical impedance spectroscopy), atomic force microscopy, and electron microscopic methods are increasingly being deployed to better understand these processes. Implementing those methods at the preclinical level enables the rapid screening of new anticancer drugs with understanding of their central mechanism for cancer therapy. In this review, principles and basic concepts of new techniques suggested for metastasis, and apoptosis examinations for research purposes are introduced, along with examples of each technique. From our recommendations, the privilege of combining the bio-electrochemical and biosensing techniques with the conventional cytochemical methods either for research or for biomedical diagnosis should be emphasized.

## 1. Introduction

**Figure 1:**
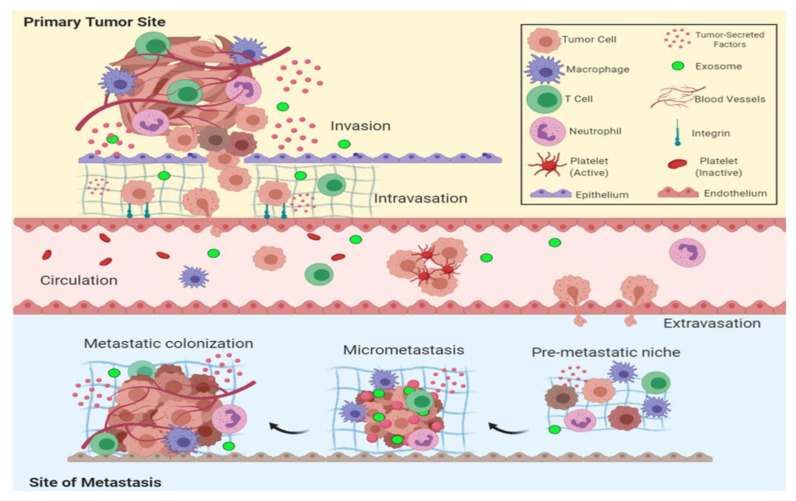
The steps leading to cancer metastasis. Initially, cancer cells breach the basement membrane and migrate across the cancer stroma. Next, intravasation into the blood vessels occurs and is followed by migration of the circulating cancer cells in the bloodstream until reaching the secondary metastatic site. Afterwards, extravasation of cancer cells through the endothelial barrier ensues; finally, colonization in the metastatic target organ forming a secondary cancer takes place [10].

Cancer is one of the leading causes of death worldwide, and has further escalated due to reduced medical care accessibility imposed by the COVID-19 pandemic-related constraints [1,2]. During cancer development, a fine line dictates whether mutated cells will propagate and grow into a vicious cancer or diminish and fade [3]. This fate is decided by the predominance of one of two opposing processes: the pro-oncogenic metastasis versus anti-oncogenic apoptosis process [4,5]. Metastasis is an essential drive for cancer progression which involves a complex stage [6]. The metastasis cascade includes the extracellular matrix degradation (ECM), the epithelial-to-mesenchymal transition (EMT) that enhances cancer cells deformability and mobility, cancer angiogenesis to increase blood supply to the cancer niche, and the enrichment of inflammatory mediators around the tumor microenvironment [7,8]. After circulating in the bloodstream, circulating cancer cells (CTCs) start to leave blood vessels, seeking a proper environment to seed, forming a dormant cell mass which transforms into an active secondary metastatic cancer [9]. The metastatic process is inefficient, facing many hurdles that malignant cancer cells must overcome. The metastasis process is demonstrated in Figure 1.

Invasion of the basement membrane is the first step in the development of a secondary metastatic tumor. This is followed by migration across the ECM. Cancer cells then enter into blood arteries and the bloodstream to extravasate to a distant secondary metastatic site due to their survival during vascular transit. Finally, colonizing cancer cells develop secondary tumors. Apoptosis, or programmed cell death, is one of the major dangers preventing cancer development [10,11].

**Figure 2:**
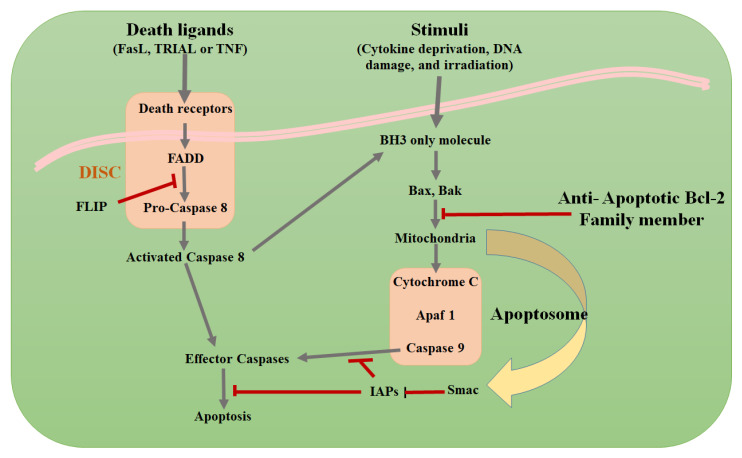
Overview of apoptosis signaling pathways and the effects of pro-survival signaling, immune cells and the tumor microenvironment. (Phosphatidylserine Fas ligand (FasL), cancer necrosis factor (TNF) and TNF-related apoptosis-inducing ligand (TRAIL), FAS-associated death domain protein (FADD), death-inducing signaling complex (DISC), activated caspase 8 (also called FADD-like IL-1 converting enzyme (FLICE)), FLICE-like inhibitory protein (FLIP), pro-apoptotic B-cell lymphoma-2 (Bcl-2) family members (Bax and Bak), apoptotic protease activating factor-1 (APAF-1), second mitochondria-derived activator of caspase (Smac), and inhibitor of apoptosis proteins (IAPs).

Apoptosis plays a crucial role in various biological processes, including normal cell turnover, embryonic development, and immune system functioning and chemical-induced programmed cell death [11,12]. Apoptosis induction is one of the most exploited mechanisms in cancer treatment, thus several anticancer drugs are currently used in clinical oncology to trigger cancer cell death [13,14,15]. The signaling cascades leading to apoptosis either proceed through extrinsic, intrinsic, or performing/granzyme pathways. The extrinsic pathway is initiated via the stimulation of surface death receptors by their cognate ligands Fas ligand (FasL), cancer necrosis factor (TNF) and TNF-related apoptosis-inducing ligand (TRAIL) [16]. After the receptor/ligand interact, FAS-associated death domain protein (FADD) and death-inducing signaling complex (DISC) become activated, leading to the stimulation of a cascade of caspase proteins, ultimately resulting in cell-death. On the other hand, different intracellular stressors can lead to the activation of the pro-apoptotic BCL2 protein family members, namely BCL2-associated X protein (BAX) and BCL-2 antagonist or killer (Bak), which oligomerize, bind to and permeabilize the outer mitochondrial membrane (OMM); thus, opening the mitochondrial permeability transition (MPT). Upon opening of the MPT, cytochrome C cofactor is released which culminates in the induction of downstream caspases and the progression of the intrinsic apoptotic pathway [17,18]. Lastly, the third pathway is activated through the stimulation of perforin, a membrane bound channel, by cytotoxic T-cells with the formation of granzyme-B. Finally, all three pathways converge at the execution step, where caspase 3 gets activated, culminating in a programmed cell death, as shown in Figure 2. A comprehensive explanation of the various types of apoptotic pathways are discussed by Cavalcante et al. [19].

At the molecular level, disrupted apoptosis underpins several human pathologies including cancer. Accordingly, the modulation of the cellular fate to induce cell-cycle arrest is utilized for its immense anti-cancer therapeutic potential [16,20]. To that end, various studies are implemented to gain in-depth understanding of both metastasis and apoptosis mechanistically. Yet, improvements in the characterization as well as the analysis techniques of apoptosis and metastasis are still needed.

In this review, we aim to discuss some of the advances relevant to the detection methods of both cancer metastasis and apoptosis. To this end, the exploitation of biophysical techniques, specifically electrochemical biosensors and atomic force microscopy, to serve as characterization tools will be discussed. Also, a comparison between such biophysical techniques and traditional biochemical techniques from a sensitivity perspective and the nature of information obtained will be highlighted.

## 2. Conventional Techniques Used to Detect Apoptosis and Metastasis Cells 

The difficult process by which initial cancer cells spread to form secondary tumors in an area of the body that is nearby or far away from their original place is known as metastasis, as was previously mentioned. To effectively treat patients with advanced cancers, early diagnosis of metastatic disease and active treatment medicines for targeting these discovered metastases remain essential. Since cancer cells need to travel distances through the blood or lymphatic system, the circulating cancer cells (CTCs) are considered to be the most reliable metastasis biomarkers. Specifically, the detection of a scarce number of CTCs requires arduous preprocessing of cell-enrichment [21,22].

Each biological process secretes or produces certain types of biomarkers that could help in early cancer detection. For example, surface epitopes of metastatic cells are employed for detection of CTCs, including epithelial (EpCAM), mesenchymal (vimentin), stem cell/EMT (octamer-binding transcription factor 4 (Oct4), Nanog, Twist), and cancer specific markers (PSA-prostate cancer) [23]. Similarly, apoptosis has distinctive biomarkers including intrinsic pathways (caspases, cytochrome- C, externalized phosphatidylserine, Fas/FasL, Bcl-2 and p53) [24,25].

On the other hand, cell morphology (metastatic and apoptotic cells) differs from normal cells, which can be trailed, distinguished, and considered for the real time identification. The metastatic cells vary in their shape according to its origin parameter that can be studied to identify the primary site of cancer [26]. Likewise, apoptosis morphological markers include chromatin condensation phase, uniformly dense nuclei, extensive plasma membrane blebbing followed by karyorrhexis, and separation of cell fragments into apoptotic bodies by “budding” [27,28,29].

Since the understanding of both metastasis and apoptosis is vital for providing an effective cancer treatment, a wide array of classical methods have been developed, modified, and applied to efficiently achieve this goal. These diverse techniques are classified into several major sub-groups mainly morphology-based techniques, biochemistry-based techniques, immunology-based techniques, and array-based techniques. Several methods are highlighted in Table 1, a list of conventional metastasis and apoptosis detection assays and their associated sensitivities have been summarized [30,31]. 

On the other hand, RECIST which stands for the Response Evaluation Criteria in Solid Tumors was considered as one of the conventional methods for the investigation of drug responses in solid tumors [32]. Imaging using the RECIST, size, and cancer cell morphologic changes could be evaluated, and based on that the therapeutic efficacy could be identified [33].

In addition to the aforementioned classical techniques, other biochemical and biophysical approaches such as biosensors, bio-electrochemical methods (cyclic voltammetry and electrochemical impedance spectroscopy), atomic force microscopy, and electron microscopic methods are recently prevailing, as they can provide complementary information for identification, characterization, and quantitative analysis. Most of the studies implemented to evaluate apoptosis induction in cancer cells are concerned with drug treatment effectiveness.

## 3. Biosensors for Cancer Cells in-vitro Assessment 

The area of translational medicine is constantly working to develop new methods. These will allow for early cancer detection, accurate cancer staging, and the assessment of a tumor’s response to chemotherapy. Therefore, for routine clinical applicability, such instruments are essential. In the investigation of new metastatic cancer features and evaluation of the efficacy of antiproliferative/cytotoxic drugs, biosensors are crucial tools in the field of cancer biology [49,50]. Essentially, electrochemically based biosensors operate through the detection of a specific biological analyte (whole cells, proteins, nucleotides, and metabolites) with subsequent conversion of the obtained signals into proportional electrical signals for further analysis via transducers [51]. Amongst a wide array of available transducers, electrochemical biosensors are going to be the focus of the review.

**Figure 3:**
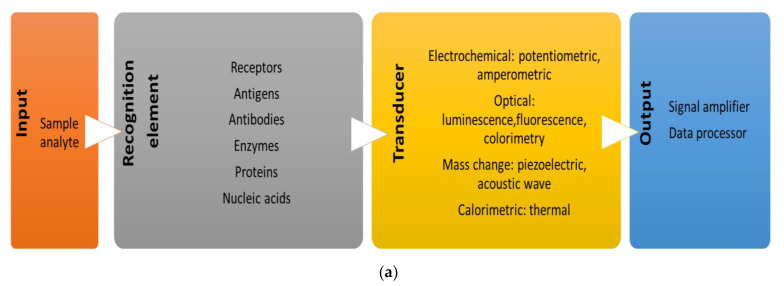

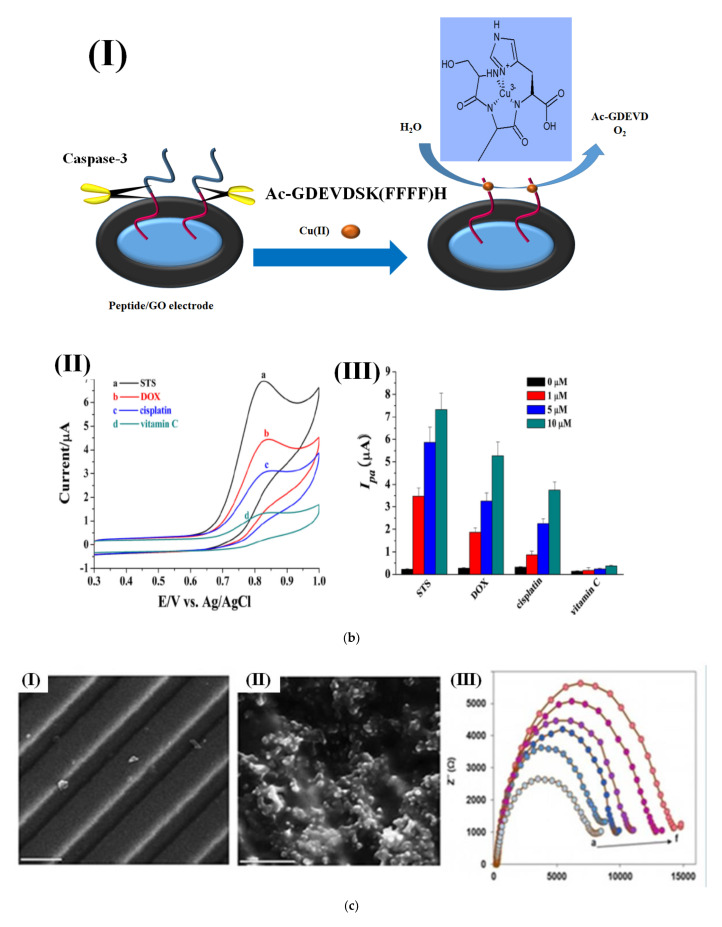

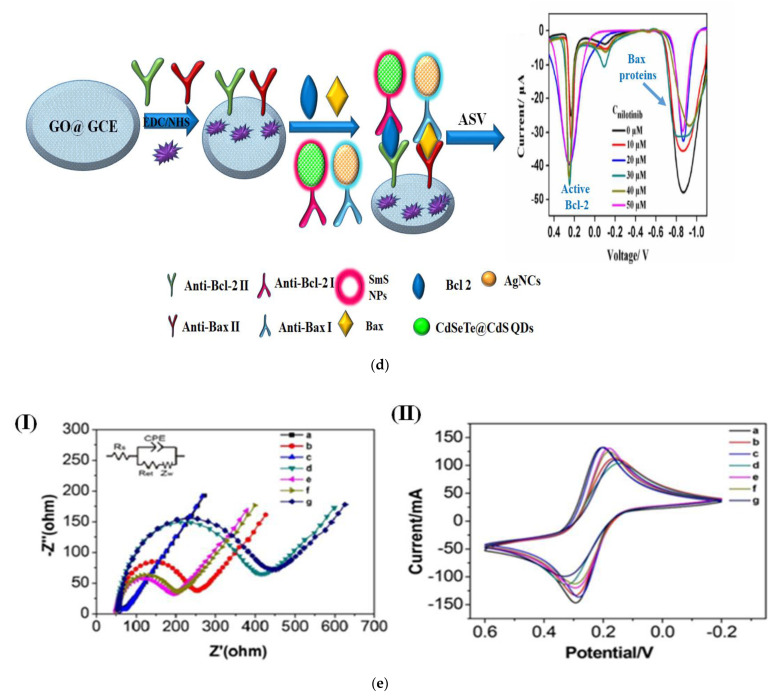

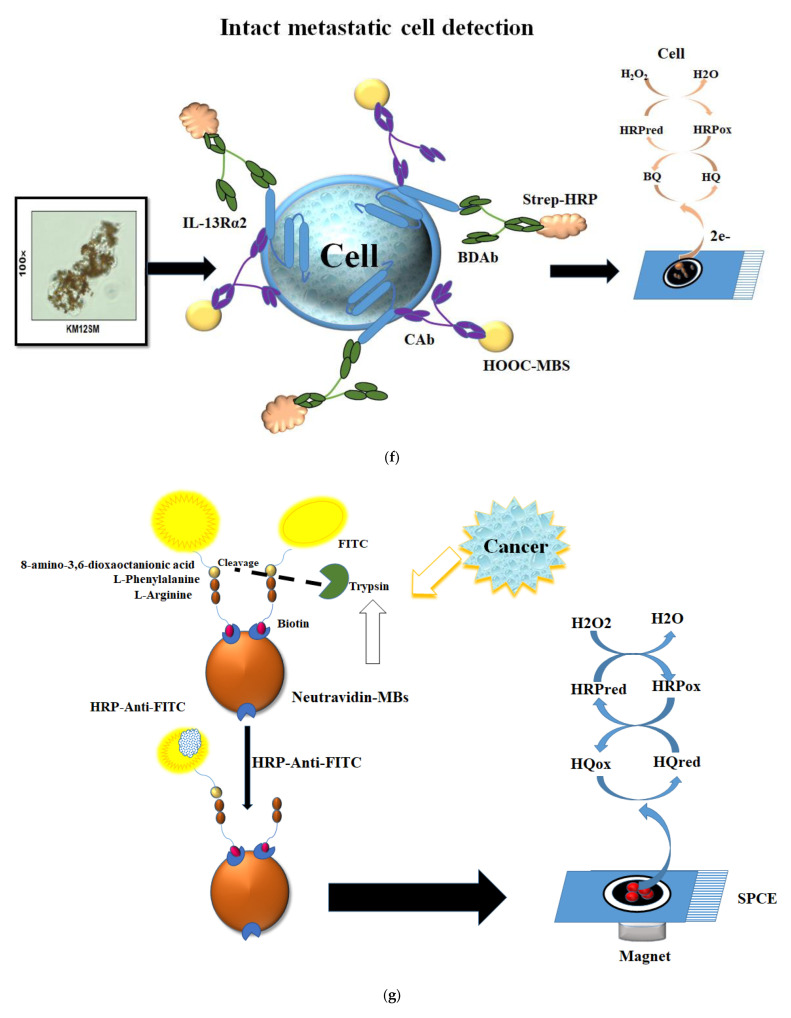
(**a**) The important components of a designed biosensor. A single or a multiple bio-receptors (could be whole cells, microorganisms, enzymes, or antibodies). A transducer of the physicochemical signals resulted from the analyte and bio-recognition elements interaction(s). Data processor to interpret and amplify the results that have been converted [52,54]. (**b**) Schematic representation of Caspase-3 electrochemical biosensor. (I) Cas-3 detection occurs through electrocatalytic activity by the cleavage product ATCUN-Cu. (II) cyclic voltammograms of Cas-3 activity when the Hela cells were incubated with 5 μM of individual anticancer agents. (III) The average of the oxidation current generated from the Cas-3 in treated HeLa cells with different concentrations of the four cancer drugs. (**c**) Aptasensor representative images and impedance measurements. The SEM images of (I) bare Au electrode and (II) Au-Apt@AgNCs modified electrode. (III) Nyquist plots for Au-Cys-Apt@AgNC after incubation with different concentrations of Cyt-C. (**d**) Schematic representation of the dual-signal-marked electrochemical immunosensor. Anti-Bax II and Anti-Bcl-2 II recognition antibodies are immobilized on glassy carbon electrodes (GCE) for the recognition of their cognate proteins in the sample. Subsequently, a signal is detected upon the formation of an immune-sandwich with the QD-modified primary antibodies anti-Bax I and anti-Bcl-2 I. (**e**) Voltammetric and impedimetric determination of epithelial cell adhesion molecule (EpCAM). (I) The Nyquist impedance spectra of the gold electrode modified at different stages in the presence of PBS containing 5 mM [Fe(CN)_6_]^4−/3−^ and 0.1 M KCl. Inset is the equivalent circuit model used to fit the impedance data. (II) Typical voltammetric measurements of the following conditions (a) Bare Au electrode; (b) MPA/Au electrode; (c) G6 PAMAM modification; (d) G6 PAMAM-COOH/MPA/Au electrode; (e) post-Anti-EpCAM addition; (f) post blocking the non-specific binding site by BSA; (g) after exposure to Hep-G2 cells (1.0 × 10^6^/ml) [66]. (**f**) Schematic demonstration of the IL-13Rα2 sandwich immunosensor. Functionalized recognition microbeads specific to IL-13Rα2 are introduced in-vitro to KM12SM metastatic cells. This leads to an immunocomplex formation. Consequently, certain reactions occur which provoke the amperometric transduction of signals. “The figure has been adapted with permission from Ref. [69]. 2022, Springer”. (**g**) Electrochemical biosensing approach for the electron-mediated determination of a metastasis-linked protease in pancreatic cancer cells. Neutravidin-MBS is linked by a biotin linker to synthetic peptide chains terminating with fluorescein isothiocyanate (FITC). The low amount of attached FITC after cleavage of his trypsin in cancerous pancreatic cells results in low amperometric response and vice versa in case of healthy cells [69].

A fully functional biosensor is mainly composed of a recognition element that detects a certain molecular component in the sample under investigation. Next, the recognition event is detected via the deployment of different transducers (electrochemical, optical calorimetric, and mass change) which relays various signals to be further amplified and processed for data analysis [52]. A brief on the main components of biosensors is depicted below, Figure 3a.

Electrochemical biosensors can be subdivided in either potentiometric or amperometric biosensors. Potentiometric biosensors employ ion-selective electrodes to sense the changes in electrical potential upon molecular recognition. While amperometric transducers measure current that is formed when a potential is applied on two electrodes. To this end, several biosensors including immunosensors, electro-chemiluminescence biosensors, impedance-based sensors, genosensors, as well as the microfluidic platforms have been developed [53,54]. The advantages of the electrochemical sensing techniques are their cost effectiveness, small sample requirement, reproducibility, fast detection, extreme sensitivity, ability to miniaturize, and user-convenience [55,56]. This section of the review is devoted to research advances in developing such electrochemical-based sensors for cancer apoptosis and metastasis biomarkers.

Since the apoptotic pathway functions through either intrinsic or extrinsic signaling with a plethora of molecular effectors. Caspase-3 (Cas-3) is considered as a crucial mediator in the extrinsic apoptosis signaling cascade that activates downstream caspases leading to cell-death. Accordingly, Yu et al. reported the use of organic electrochemical transistors (OECT) to detect Cas-3. The design was based on gold–sulphur bonds-mediated anchoring of the Cas-3 recognition sequence Asp-Glu-Val-Asp (DEVD) onto the modified glassy carbon electrode with gold nanoparticles. The obtained results demonstrated that the transfer curve of the transistor was shifted to a lower gate voltage post-surface modification using peptide monolayer. To investigate the biosensor performance, Cas-3 detection was implemented in aqueous solutions where the obtained data proved a high sensitivity, whereas the limit of detection was 0.1 pM. In addition, at the cellular level using Hela cells, the developed sensor could efficiently determine 10 apoptotic cells/10 μL [57].

Similarly, Dehua Deng and collaborators developed an electrochemical sensing platform to determine the activity of Cas-3 in Hela cervical cancer (HCC) cells pre-incubated with four different cytotoxic agents [58]. In that study, graphene oxide (GO)-modified electrodes were functionalized with a peptide substrate specific to Cas-3 to be utilized as bio-receptors, as shown in Figure 3b. 

When the Cas-3 cleaves the peptide substrate, the amino terminal Cu(II) and Ni(II)-binding (ATCUN) motif on the electrode surface is exposed. The ATCUN Cu(II) complex has a good electrocatalytic property to enhance and accelerate the electrochemical signals. The results showed that the generated electrical current reached its maximum upon treatment with 5 µM of staurosporine > doxorubicin > cisplatin > vitamin C. As the concentration of Cas-3 increases, the current increases with detected concentration range of 0.5 pg/mL–2 ng/mL, as depicted in Figure 3b [59].

Another aspect of detection, the initial step in the cascade of intrinsic apoptosis pathways, is the release of cytochrome-c (Cyt-c) from the mitochondrial to the cytosol with subsequent stimulation of downstream caspases. Based on this, Mojtaba Shamsipur and collaborators exploited electrochemical impedance spectroscopic techniques (EIS) to develop a sensitive aptasensors specific for Cyt-c using silver nanoclusters (NC) conjugation (Au-Cys-ssDNA@AgNCs) [60]. Utilizing HEK293T human embryonic kidney cells, the EIS-generated signals were found to be dependent on the release of Cyt-c concentration (Figure 3c). Accordingly, the concentration of Cyt-c was quantified by measuring the increase in the interfacial charge transfer resistance (R_ct_) where the NCs were immobilized on gold electrodes to enhance detection sensitivity to reach a range of 0.15–375 nM with the limit of detection of 72 pM. Such sensitivity was attributed to the increase of the surface to volume ratio due to using Ag-NCs (the active bio-sensing layer) which resulted in high surface density of the aptamer units (recognition moieties). It is worth-mentioning that EIS is one of the most convenient *in-situ* electrochemical analytical techniques as it requires no further labeling of redox active moieties. Moreover, aptamers, which are synthetic oligo-nucleotide sequences, maintained higher stability in bioanalytical assays compared to other bio-recognition moieties, such as antibodies and oligopeptides.

Normally, cells exert control over the apoptosis process through a class of Bcl-2 family regulator proteins. Conventionally, in cancer research the ratio between the Bcl-2 (anti-apoptotic) and Bax (pro-apoptotic), protein expression is used to evaluate the apoptosis degree upon varying drug dosage and incubation time. The effectiveness of nilotinib in inducing apoptosis in chronic myeloid leukemia K562 treated cells was investigated using a dual-signal electrochemical immunosensor which is developed by Shiwei Zhou and collaborators. This biosensor was composed of glassy carbon electrode (GCE) coated with reduced graphene oxide (RGO) on which target recognition Anti-Bcl-2 and Anti-Bax antibodies are immobilized, Figure 3d [61]. Subsequently, the immunosensor was exposed to Bcl-2 and Bax active antigenic proteins and then, the electrode was further exposed to targeted NCs coated by antibodies (signal probes). The resultant sandwich electrochemical immunosensor was in-turn washed with an acid solution to produce the corresponding Ag^+^ and Cd^2+^ ions that are assessed by anodic stripping voltammetry (ASV). The produced voltammetric signal is used to detect the concentration of the electrode bound antigens. The developed biosensor reached a limit of detection of 1 × 10^3^ cells, which is more sensitive than the reported conventional methods [61].

Unlike the previous studies where the protein product is the target moiety, Li and co-authors developed an antibody-based microfluidic microchannel device to detect the intracellular mRNA levels of an antiapoptotic biomarker called Survivin (Sur) [62]. The microchannel cytosensor could selectively capture prostate cancer cells through recognition of prostate stem cell antigen (PSCA) by attachment of cognate monoclonal antibodies. Then, recognition of intracellular Sur mRNA recognition was done using graphene oxide-based nanocarrier tagged with an anti-survivin oligonucleotide sensor. The genosensor is further labeled by fluorescein isothiocyanate to be able to act as a signal nanoprobe which is able to detect (4.8 ± 1.8) × 10^6^ copies/cell. Survivin was also reported to be detected in circulating cancer cells (CTCs). Therefore, the analysis of CTCs can provide alternative non-invasive diagnosis of cancer metastasis and recurrence [62].

Another label-free micro-device was developed that relies on CTCs physical properties rather than biological affinity to operate. Compared to RBCs, CTCs are considered larger in size with lower deformability. This approach can isolate viable cancer cells from blood of lung, breast, and colon cancer patients [63]. This was preceded by the innovative attempt by Nagrath and co-authors, who developed the ‘CTC-chip’, which performs affinity isolation of viable CTCs utilizing anti-EpCAM-coated micro-spots under controlled laminar flow conditions. The device was reported to provide a selectivity and sensitivity comparable to immunomagnetic beads [64]. It is worth mentioning here that the CTC-derived ex-vivo cultures are essential to identify the role of CTCs in metastasis, and to understand the CTCs biology to eventually provide a means for personalized cancer drug testing, and disease surveillance [65].

In another recent study, Jianguo Xu and collaborators developed an impedimetric-based immunosensor for detection of metastasis-related a trans-membrane protein known as epithelial cell adhesion molecule (EpCAM) [66]. The assembly layer is made of a gold electrode functionalized with polyamidoamine dendrimer (G6-PAMAM) which in-turn is terminally attached to EpCAM bio-sensing antibody. Upon specific binding of the antibody to EpCAM antigen, an electrochemical signal is generated that could be accurately quantified by the voltammetry and electrochemical impedance spectroscopy (EIS), Figure 3e. Upon biological application using Hep-G2 hepatic carcinoma cells, a limit of detection of 2.1 × 10^3^ cells/mL was achieved [66].

In another biosensing report introduced by Alejandro Valverde and collaborators, colon cancer metastasis was electrochemically diagnosed through the development of a novel immunosensor to amperometrically monitor the secreted IL-13Rα2 as the targeting cancer biomarker [67]. The immunosensor is made of antibody-modified magnetic microbeads (MBs) for the selective capture of IL-13Rα2 and biotinylated detection antibodies (BDAb). The BDAb were labeled by streptavidin-horseradish peroxidase (Strep-HRP) as a probe, Figure 3f. In order to recognize the affinity reaction, the sandwich immune-complexes were magnetically loaded onto disposable carbon screen-printed electrodes (SPCEs) using the (H_2_O_2_)/hydroquinone (HQ) system which is assigned for the amperometric measurements. The developed immunosensor was tested for *in-situ* quantification of IL-13Rα2 expression in both lysed and intact colon cancer cells. Compared to the traditionally used immunochemical assays, the designed immunosensor proved to be faster than ELISA, saving more sample than western blot, and very sensitive, with the limit of detection of 1.2 ng/mL. the authors concluded in their report that this kind of biosensor can be used to assess the metastatic potential of cells [67]. 

Besides the accurate and rapid inspection of apoptosis, metastasis was tracked and studied by electrochemical biosensors. For example, aptasensor was constructed to recognize L-Tryptophan (Trp) based on constant current-potentiometric striping analysis, as shown by M. R. Majidi and co-authors [68]. The excess metabolism of Trp indicates the presence of metastatic cancer cells which helps in suppressing the immune system and enhances metastasis progression. This aptasensor consisted of multiwall carbon nanotubes (MWCNTs) fixed on a gold electrode, then attached to the L-tryptophan-Aptamer. The aptasensor proved to be sensitive with the LOD of 6.4 × 10^−11^ M. Also, the metastatic potential of different cancer cell lines was successfully discriminated based on Trp consumption rate [68].

On the other hand, metastasis of pancreatic cancer cells was monitored electrochemically via biosensing the expression of trypsin in cell lysate, which changes its level in cases of pancreatic cancer [69]. The invasion as well as metastasis of pancreatic cancer is regulated by aid of trypsin. Hence, the biosensor was made of Neutravidin-MBS linked to a synthetic chain of peptide by biotin linker. This peptide chain was terminated with fluorescein isothiocyanate (FITC). In the presence of a high percentage of trypsin, such as in cancerous pancreatic cells, which cleaves the bond between the FITC and the peptide, the low amount of the attached FITC resulted in lowered amperometric response and vice versa in the case of healthy cells, Figure 3g demonstrated the construction steps of the biosensor used for the reported case [69].

Another electrochemical biosensing for the simultaneous detection of the metastatic biomarkers programmed death ligand-1 (PD-L1) and hypoxia-inducible factor-1 alpha (HIF-1α) was developed by Muñoz-San Martín and co-authors [70]. The biosensor design is based on target proteins being captured on antibody-modified carboxylic magnetic microbeads (MBs) followed by enzymatic labeling using horseradish peroxidase (HRP)-labeled antibodies. Afterwards, amperometric detection takes place at disposable screen-printed carbon electrodes (SPCEs) relying on the hydrogen peroxide/peroxidase/hydroquinone system. Sandwich immunoassays were executed for both the biomarkers and the fabricated sensors were ultrasensitive achieving LOD values of 86–279 pg/ mL [70].

Lastly, cancer cell viability and cellular microenvironment were investigated electrochemically using 2D and 3D-sensing cell culture Flask (SCCF) integrated with an electrochemical station for recording the obtained electrochemical signals due to metabolic activity, and/or extracellular changes. Simply, nano- or micro-fabricated sensor chips are fixed in standard cell culture flasks to allow the electrochemical inspection of the cell’s activity during the cultivation and growth cycles [71]. Thus, such electrochemical cancer-on-a-chip biosensing systems have the potential to become crucial tools for the study of cancer development and drug efficacy.

As reported, such systems have been applied for measuring the electrochemical behaviors of brain cancer (T98G) and breast cancer (T-47D) cells. In addition, amperometric oxygen sensors were used to screen cellular respiration with different incubation conditions. Moreover, cellular acidification was accessed with potentiometric pH sensors using electrodeposited iridium oxide films. The system itself provides the foundation for electrochemical monitoring systems in 3D cell culture [72].

To summarize the roles of electrochemical biosensors in cancer metastasis and apoptosis detection, the simple design of biosensing devices makes them a reliable tool for the early detection of specific biological targets by converting a biological entity (cell viability, protein binding affinity, DNA or RNA sensing) into an electrical signal that can be measured and analyzed. Particularly, in cancer detection and monitoring, biosensors hold great potential. Thus, biosensors could be easily designed to monitor emerging cancer biomarkers and to evaluate the drug effectiveness at various target sites. Eventually, biosensor technology has the potential to provide fast and accurate detection, reliable imaging of cancer cells, and monitoring of angiogenesis and cancer metastasis, and the ability to determine the real-time continuous responses of cancer or normal cells to discover potential, sensitive, and active anticancer chemotherapy agents. The future directions for cancer electrochemical sensors designs are going to be focused on the development of new functional polymeric substrates (biocompatible, and flexible chips) for effective cell adhesion and proliferation, or nanostructured biomaterials to be valid for nano-and microfabrication techniques. On the other hand, sensing elements incorporated in 2D cultures and 3D scaffolds could be utilized to facilitate advanced organ-on-chip studies in the future as well as for improving micro-scale understanding of cell culture microenvironment [73].

## 4. Atomic Force Microscopy (AFM) Utilization in Cancer Cells Investigation

Mechanics are intrinsic properties that appear throughout the formation, development, and aging processes of biological systems. Mechanics have been shown to play important roles in regulating the development and metastasis of tumors and understanding cancer mechanics has emerged as a promising way to reveal the underlying mechanisms guiding cancer behaviors. Thus, investigating cancer mechanics on multiple levels is significantly helpful for comprehensively understanding the effects of mechanics on cancer progression [74,75,76].

**Figure 4:**
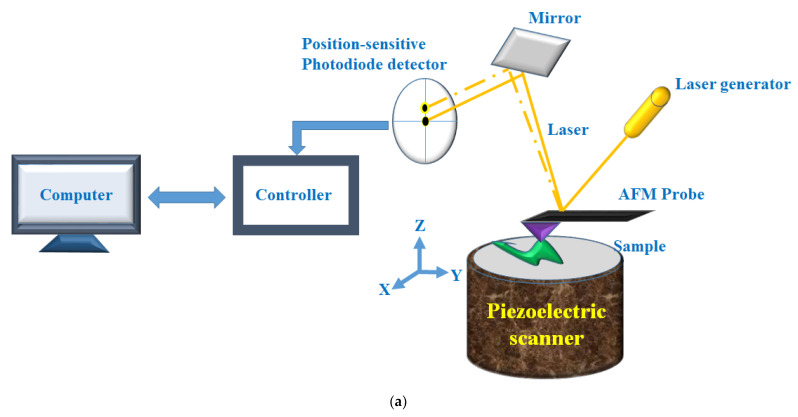

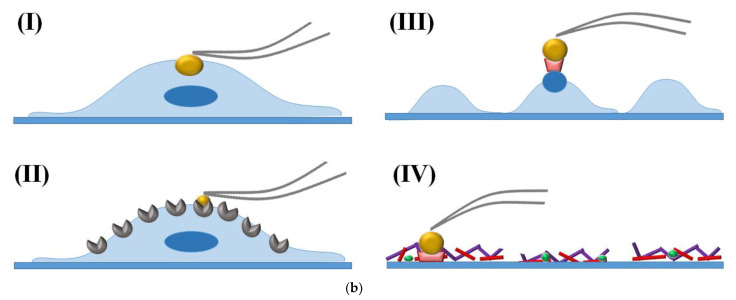
(**a**) Schematic diagram of AFM working principles. The AFM instrument is composed of a main probe (cantilever), a laser source, a piezoelectric material, and a quadruple photodiode detector. The AFM probe is a flexible cantilever with a sharp micro-tip that is attached at its end. The tip, which has a monomolecular point, allows for nanometer resolution imaging and the micro-cantilever is a force sensor that can detect even minute deformation of a sample, enabling very high sensitivity AFM in force measurement. (**b**) Schematic illustrations of the AFM-based biomechanical assays. (I) Indentation experiments were used to characterize the compliance of a single cell. (II) Single molecule force spectroscopy assesses interactions between a functionalized probe and the membrane receptors. Single-cell force spectroscopy was utilized to quantify (III) cell–cell adhesions and (IV) cell–substrate adhesions.

Thus, direct live-cell imaging/analysis for the morphological dynamics with nanometer resolution under physiological conditions is needed. In this regard, atomic force microscopy (AFM) that provides ultrastructural details with atomic resolution under near-physiological conditions could be applied in cancer research [77]. AFM is an extremely high-resolution tool (a horizontal resolution of 0.1 nm, a vertical resolution of 0.01 nm) to be effectively used to investigate the morphological/topological structures of specimens, and quantitatively measure its mechanical properties at atomic resolution [76]. AFM was explored in 1986 by G. Binnig, C. F. Quate, and C. Gerber at Stanford University. Fundamentally, the AFM uses a physical probe to sense the morphology of the sample under investigation in three-dimensional space, obtaining information from the very weak atomic interaction between the probe-tip and the atoms of the sample surface. The rapid and widespread adoption of the AFM in cancer biology refers to its technical advantages that could be summarized as follows: high resolution with the possibility to perform direct three-dimensional imaging of molecular and even atomic-scale structures (Figure 4a). Under near-physiological conditions, dynamic processes of molecules, organelles, and other structures in living cells can be monitored in real time. The functionalized probe-tip can be used to identify specific molecules or interaction forces such as ligand-receptor interactions. For all of these reasons, AFM could be incorporated within the analytical approaches for cancer in the biomedical field. 

Mechanical phenotyping of cells by AFM was proposed as a novel tool in cancer cell research, as cancer cells undergo massive structural changes comprising remodeling of the cytoskeleton and changes of their adhesive properties. To this end, application of non-functionalized AFM tips is used to obtain mere topographical information about the dynamic states and mechanical changes of live cells. Yet, through the tip modification, the scope of use is extended to include molecular interactions via detection of individual ligand-receptor interactions. Moreover, tips of the AFM probes can be replaced by coated microspheres to induce the formation of focal-adhesion-like structures to study cell adhesion capabilities [78]. Depicted in Figure 4b, there are different parameters related to metastasis that can be studied using AFM. Recently, great advances have also been introduced to examine subcellular structures using multi-harmonic AFM techniques as well [79,80].

The AFM enables researchers to study cell stiffness and deformability, which is relevant to metastatic/migration potential of cancer cells through investigating Young’s modulus value (ε). This mechanical property correlates strain (proportional deformation) caused to a cell after being exposed to stress (force per unit area) applied by AFM probe tip. Thus, the lower the obtained value, the higher the cells’ deformation capability. Also, force-displacement curves are deployed to quantitatively assess a material’s elastic modulus, adhesion, and stiffness [76].

**Figure 5:**
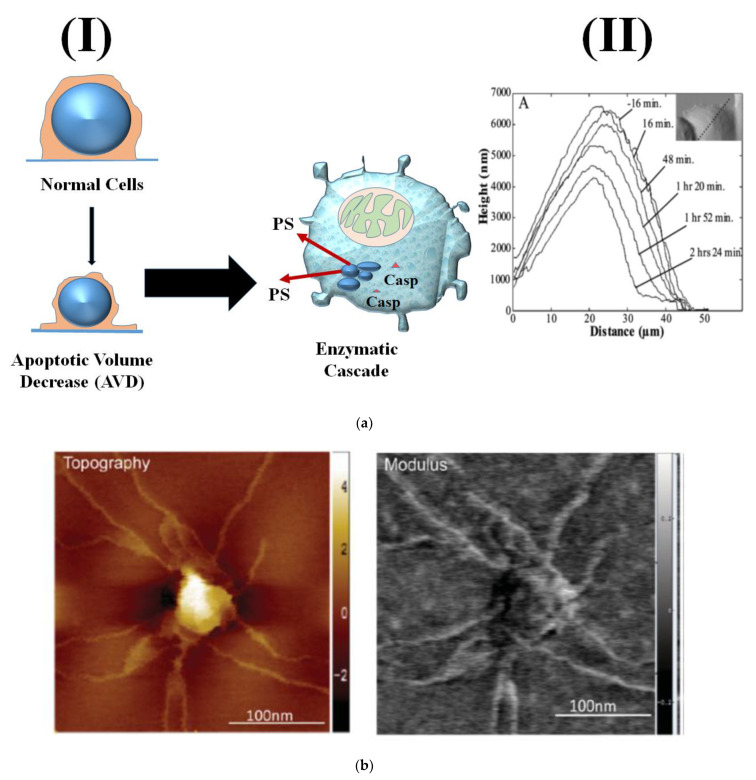
(**a**) Biophysical assessment of apoptotic volume decrease (AVD) in staurosporine treated cancer cells. (I) schematic depiction of biophysical and biochemical changes taking place during apoptosis chronologically. (II) Line scans for AFM images of KB cells exposed to 1 µM staurosporine (STS). Inset shows line scans taken across dashed lines. (**b**) AFM images depict GBM U87-derived exosomes with multiple nano-filamentous structured surface protrusions. Quantitative mapping of several biophysical parameters, including topography (range z = 5 nm) and modulus (z = 2 GPa) are presented.

An early study by Hessler and collaborators combined AFM, microscopy (light and fluorescence), and flow cytometry techniques to quantify time-dependent cellular morphological changes associated with staurosporine-induced apoptotic volume decrease (AVD) [81]. Contact-mode AFM images showed that AVD occurs uniformly throughout the nucleus and the cytoplasm in treated KB human epidermoid carcinoma cells, Figure 5a. Importantly, AFM imaging proved superior, as the resultant morphological changes, especially at the cellular edges, were not visible when employing standard light microscopy techniques. The integration of confocal microscopy post nuclear staining showed that during AVD the nuclear integrity remained unaltered. Moreover, flow cytometric data analysis confirmed the hypothesis of temporal precedence of AVD followed by biochemical changes. This study exemplifies the importance of combining various biophysical and biochemical techniques to harvest comprehensive multi-parameter information about the cellular events [81].

In accordance with the previous results, a recent study reported that during apoptosis the biomechanical alterations and geometric reconstruction are considered more sensitive biomarkers than biochemical signals. To this end, cervical cancer HeLa cells induced by cyto-chalasin B (CB) were used as an *in-vitro* model. Employing mechanical mapping, AFM revealed a noticeable decline in cellular elastic modulus as well as volume, accompanied by an increase in surface roughness prior to the activation of apoptosis signaling cascade [78]. Moreover, confocal fluorescence visualization demonstrated that actin filaments depolymerization induced the reorganization of membrane proteins and membrane blebbing. Actin disassembly was inversely proportional with the cellular elastic modulus and volume yet directly proportional with surface roughness and CD95/Fas activation [82].

Likewise, the re-organization of cellular cytoskeleton promotes metastasis via imposing vast changes on the cell–cell adhesion and dynamics which in-turn facilitates invasion into neighboring tissue, and intra- and extravasation into lymphatic or blood circulation. Accordingly, the biomechanical properties of several human cancer cell lines having different metastatic potential were assessed using AFM-based microrheology experiments. Using this vasoelastic mapping technique, quantification as well as distinction in mechanical properties of cancerous cells from the malignancy perspective was done. Through, measuring loss of tangent (η = G″/G′), the ratio of loss modulus to storage modulus of the probed cell, the energy dissipated upon cell deformation at different frequencies was quantified. The loss tangent is inherently model-independent and conveys information as to whether a cell is rather solid or fluid at a given excitation frequency. It was concluded through the force maps that cellular fluidic-behavior is directly proportional with the metastatic potential of cells from MCF-10A, representing benign cells to highly malignant MDA-MB-231 cells [83].

Another metastatic biomarker that is gaining increasing attention recently is exosomes. Exosomes are known to play an important role in cancer proliferation, intracellular communication in pre-metastatic niches, as well as tumor microenvironment modulation [84]. Their surface molecular composition and dynamics using glioblastoma (GBM) has been studied by Sharma and collaborators, Peak force microscopy (PFM) images revealed distinctly stiffer and more adhesive nano-filaments extending from the surface of GBM exosomes, depicted in Figure 5b. Size of U87 exosomes measured from typical high-resolution PFM (HRPFM) topographic images is approximately 89.3 nm in diameter and 4 nm in height. Those tentacle-like nanofilaments have been proven to enhance the cellular uptake of the exosomes [85]. 

**Figure 6:**
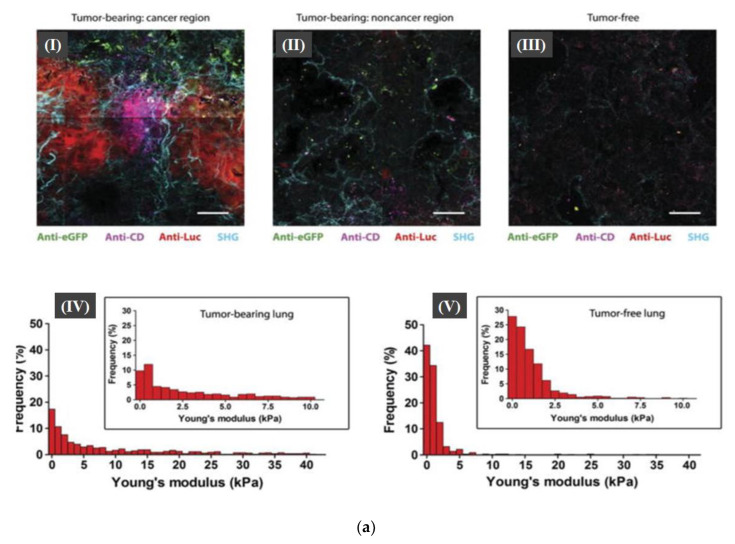

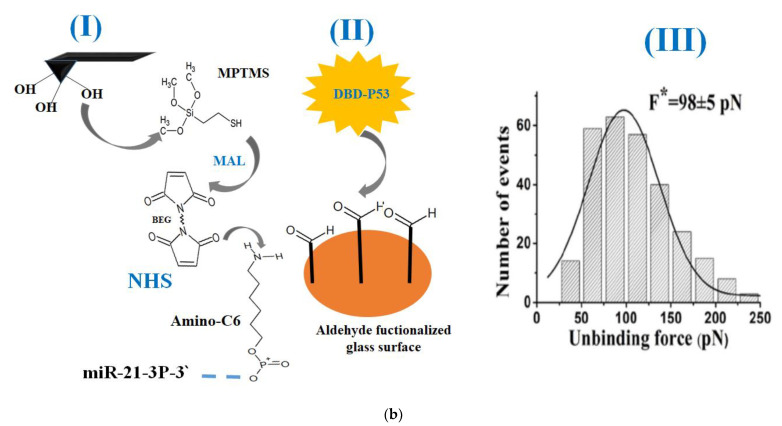
(**a**) Specific activation of MRCS in response to mechanical cues in the metastatic niche *in-vivo*. Frozen lung sections of tumor-bearing NSG mice cancer region (I) and noncancer region (II) and tumor-free NSG mice (III) after infusion of MRCS-CD cotransfected with eGFP were stained with anti-Luc (red) to detect lung metastasis, anti-CD (magenta) for CD expressed by MRCS-CD, and anti-eGFP (green) for MRCS-CD tracking. SHG imaging of collagen networks (cyan) was also overlaid on IHC imaging. (IV) and (V) Frequency of Young’s modulus values of cancer bearing and tumor-free (VI) lungs from AFM microindentation in the range of 0 to 40 kPa (bin size = 1 kPa), whereas the inset graphs show the frequency within the range of 0 to 10 kPa (bin size = 0.5 kPa). The Figure is adapted with permission from Ref. [87]. 2022, Elsevier. (**b**) AFM technique deployed to investigate the molecular interaction between the pro-oncogenic miR-21-3p and the tumor suppressor p53. Schematic depiction of the surface chemistry used to covalently bind (I) miR-21-3p and DBD of p53 (ii) to AFM tips and substrate, respectively. (III) SMFS histogram of the unbinding forces for the DBD-miR-21-3p complex [87].

From the abovementioned studies, it was evident that combining optical microscopy with AFM gives comprehensive spatiotemporal information with high biochemical specificity. The high spatial resolution is attributed to AFM, while the precise temporal/biochemical sensitivity is obtained through the integration of fluorescence-labeling based optical microscopy. The fluorescence labeling might impose some technical difficulties. Thus, recently Liu et al. suggested the use of a mechano-responsive cell system (MRCS) that responds to mechano-environmental stimulations, which in-turn localize and target therapeutics specifically to metastatic niches. This mechanosensitive promoter-driven mesenchymal stem cell (MSC)-based vector was proven to effectively kill cancer cells as demonstrated in a metastatic breast cancer bearing animal model. Utilizing label-free second harmonic generation (SHG) imaging, cancer metastasis was found to be associated with high collagen expression, Figure 6a. In addition, observed collagen networks were found to be more linearized in cancerous niches relative to noncancerous neighboring areas. On the other hand, mechanical AFM revealed an increased heterogeneous stiffness in cancer regions. Conclusively, the combination of AFM and SHG findings indicated that metastatic cancer niches acquire a distinctive mechano-environment that can be used as a diagnostic as well as therapeutic target. This study underscores the power of AFM tip functionalization in detecting cell–cell interaction, a technique called single-cell force spectroscopy [86].

Like SCFS, Single-molecule force spectroscopy (SMFS) can directly measure molecular interactions at the surface of viable cells in a more time- and cost-efficient manner to detect intermolecular interactions, compared with traditionally used methods such as radioimmunoassay, surface plasmon resonance (SPR), and fluorescence resonance energy transfer (FRET). Thus, integrating SMFS analysis data can enhance discovering novel cancer biomarkers drug receptor interactions. This is best demonstrated in a recent study by Moscetti and co-authors to study the in-vitro molecular interaction between the pro-oncogenic miR-21-3p and the tumor suppressor p53 [87], Figure 6b. This interaction is proposed to block the DNA binding domain (DBD) of p53 to hinder its anti-apoptotic activity to test this hypothesis DBD is linked to a fluorescence label. Using SMFS, specific high affinity interaction (10^5^ M) was proven to take place through the approach-retraction cycles. While FRET results suggested that p53 inhibition might occur via interaction with Trp-146 in-turn blocking either DNA binding or p53 oligomerization events [87].

Through other recent reports, lipidic monolayer in pulmonary epithelial cells was imaged by the AFM after the exposure to benzolconium and cetylpyrimidinium chloride (BAC, CPC). Deposition of disinfectant on the lung surface alters the alveolar surface activity, thus the AFM showed that cell death resulted from the caspase-3-dependant pathway and the topographical changes resulted from a reduction of surface compressibility, a change in the isotherm shape, and a decrease in the size of the condensed lipid domains resulted from BAC and CPC [88].

The successful development of cancer-specific biosensors relying on the incorporation of selective biomarkers can be utilized as a diagnostic tool with clinical applicability. This specifically can be an interesting topic to be investigated. Moreover, for research purposes, high resolution of 3D images with sub-nanometer-scale could be provided by the AFM to study the ultrastructure and mechanical properties of tumor cells.

Thus, the utilization of AFM with other techniques to reveal the cellular complexity would provide insightful information that would differentiate between cancer types and assess their mechanical properties. 

## 5. Conclusions, and Future Perspectives

Apoptosis is a vital process for normal eukaryotic development as it contributes to the renewal of cells and tissues, and it plays a role in the removal of needless cells through phagocytosis and prevents undesirable immune responses. Various studies (functional, biochemical, and morphological analysis) are implemented to gain in-depth understanding of both metastasis and apoptosis. Yet, improvements in the characterization as well as the analysis techniques of apoptosis and metastasis are still needed. Thus, this review highlighted a variety of biophysical and bioelectrochemical modalities (such as cyclic voltammetry, electrochemical impedance spectroscopy, atomic force microscopy, and electron microscopic methods) for investigating metastasis and apoptosis. However, it should be emphasized that one method shall not be sufficiently informative because each method covers a different prospect of the biological process under investigation. Thus, a multiparameter observational approach which is achieved via incorporating the concepts, and methods pertinent to cellular biophysics into traditional experimental paradigms shall be most useful. This multidisciplinary approach integrates the mechano-biological properties of living cells and applied mechanical force experiments studied by AFM into a focus on the field of cancer biology. Furthermore, combining high resolution imaging techniques like AFM and SEM, capable of providing lateral nanoscale information can help differentiate between non-cancerous and cancerous phenotypes. Finally, the advancement in electrochemical analysis techniques shall serve to produce tailored sensitive biosensors to precisely measure specific cancer biomarkers. To this end, biophysicists and bioengineers need to collaborate with cancer biologists, integrating their knowledge to solve experimental problems in order to gain insightful information of maximal physiological relevance.

## Figures and Tables

**Table 1:** A list of classical methods used for apoptosis and metastasis analysis [27,28,30,34,35,36].

Method	Number of Cells	Sensitivity	Advantages	Disadvantages
**I-In Vitro morphological evaluation of apoptosis and metastasis**				
Fluorescence Correlation Spectroscopy (FCS) [37].	Single	Single Molecule (<pM)	− Enhanced spatial and temporal resolution. − Rapid analysis time. − Little sample consumption, with cell viability being maintained. − Non-invasive.	− Difficulty in data analysis and interpretation. − High noise to signal ratio unless combined with other techniques.
Flow Cytometry (FCM) [38,39,40].	>10,000 cells yet investigated cell by cell	pM-nM	− Accurate quantitative method − Viable and fixed single cells can be used.	− Time consuming
Light microscopy [41].	Many	Max. resolution 200 nm Max. magnification 1400×	− Inexpensive. − Viable cells can be imaged. − Time effective, no sample coating is required like EM.	− Qualitative not quantitative method depending on cellular morphology. − Less sensitive at low magnification, thus prone to subjectivity.
**II-Electrophoretic analysis for the detection of apoptosis and metastasis**				
Western Blotting(WB) [42].	Cell Lysates	pM-nM	− Sensitivity − Specificity due the use of antigen specific antibodies.	− Susceptible to false results. − Expensive. − Technical Demand. − Invasive method
Gel-electrophoresis for detection of DNA fragmentation [43].	Single or several	N A-1	− Sensitive	− Procedural damage to the cell membrane might occur, altering the distribution of cells giving false positive necrotic cell population. − Qualitative rather than quantitative results. − Lengthy multi-step process makes it time consuming. − Cell viability is lost.
**III-Immunohistochemical detection of apoptosis and metastasis**				
Immunohistochemistry (IHC) [44,45].	Many	N A-1	− Fresh or frozen tissue samples can be used. − Inexpensive.	− Procedural damage to the cell membrane might occur, altering the distribution of cells giving false positive necrotic cell population. − Lengthy multi-step time-consuming techniques. − Subjective interpretation of data.
**IV-Immunology based techniques**				
Enzyme-linked immunosorbent assay (ELISA) [46].	Cell-lysates	pM	− High sensitivity − Inexpensive − Quantitative	− False results might occur due to non-specific antibody binding. − Requires a relatively large amount of samples (antisera).
**V-Array based techniques**				
Gene Expressions (mRNA) [47,48].	Extracted RNA	pg/mL	− Allows for discovery of novel proteins − Quantitative	− Time consuming. − Requires a complex protocol for sample preparation.
